# The impact of the COVID-19 pandemic on the dental-maxillofacial emergency service of a German university hospital in the year 2020

**DOI:** 10.1007/s00784-021-04010-7

**Published:** 2021-06-26

**Authors:** D. G. E. Thiem, M. Polsak, P. Römer, M. Gielisch, S. Blatt, B. Al-Nawas, P. W. Kämmerer

**Affiliations:** 1 Department of Oral and Maxillofacial Surgery, Facial Plastic Surgery, University Medical Centre Mainz, Augustusplatz 2, 55131 Mainz, Germany; 2 Department of Oral and Maxillofacial Surgery, School of Dentistry, Kyung Hee University, Seoul, Korea

**Keywords:** Government guidelines, CMFS, Primary care, SARS-CoV-2, Healthcare system

## Abstract

**Objectives:**

COVID-19 is considered one of the most serious pandemic in history and has posed major challenges to the world’s health care. Dentistry and oral and maxillofacial surgery (CMFS) are particularly affected due to direct exposure to the respiratory tract, as the reservoir of SARS-CoV-2. In this study, the impact of the COVID-19-pandemic on a dental and CMFS emergency services in Germany in 2020 was first time investigated and correlated with governmental restriction measures in public life.

**Materials and methods:**

Epidemiological data of a German University Hospital were analysed from a total of 8386 patients in 2019 and 2020. Parameters included information on demographics, time, weekday and reason for presentation, as well as diagnosis and therapy performed. Data from 2020 were compared with those from 2019, taking into account the nationwide periods of public life restrictions.

**Results:**

In 2020, 22% fewer patients presented via dental and CMFS emergency service. In a monthly comparison, there were negative peaks of up to − 41% in November, but also a plus of 26% in July. The largest decreases were recorded during the lockdown periods in spring (− 33%) and winter (− 39%). Further, a threefold increase in actual emergencies and inpatient admissions revealed during these time periods (*p* < 0.001).

**Conclusions:**

COVID-19 pandemic had a significant impact on the dental and CMFS emergency service in 2020 resulting in more severe cases.

**Clinical relevance:**

This study underlines the importance of maintaining an emergency service system and basic outpatient care in these specialities, which requires uniform recommendations from the medical-dental societies and politics.

## Introduction

In Germany, the provision of emergency dental services is regulated by the associations of panel dentists on the basis of a guarantee mandate. According to this, all dentists in private practice must participate in principle in the dental emergency service on the basis of the professional regulations of the respective applicable health professional chamber laws. In some cities with dental universities, the emergency dental service is provided by the dentists in private practice and the university hospitals. The definition of dental emergencies is inconsistent and not clearly defined. According to a German-language statement from 1994, a distinction is made between absolute (accidental injuries in the dental, oral and maxillofacial region, post-operative bleeding after dental surgery and odontogenic infections) and relative indications (all other diseases originating from the dental system with pain symptoms) for emergency therapy, which are also the most common reasons for emergency presentations in most industrialised countries [1–3]. The situation is different for emergencies in the field of oral and maxillofacial surgery, which require immediate therapy much more frequently. These include extended cervicofacial infections, fractures of the alveolar process and mandible, as well as of the central and centrolateral midface. In most cases, dental emergencies are not life-threatening but can be painful and/or cosmetically significant. Coronavirus disease 2019 (COVID-19), caused by the severe acute respiratory syndrome coronavirus 2 (SARS-CoV-2), has caused much public fear and confusion and has affected the delivery of vital health services, including dental care. In the context of the COVID-19 pandemic, the recommendations of the dental societies differed in a worldwide comparison. For example, in an interim guidance on the management of emergency and urgent dental care, on 16 March 2020, the American Dental Association (ADA) recommended treating dental emergencies only and published a triaging action recommendation [4, 5]. The Scottish Dental Society referred to an existing recommendation on emergent, urgent and standard care triage [6, 7]. In contrast, the Federal Dental Association in Germany advocated the maintenance of dental care and the implementation of preventive and therapeutic measures, taking into account appropriate hygiene standards. Timely and major reorganization of dental care services is challenging. Early management of acute dental emergencies is important to avert patients from Accident and Emergency services and to avoid hospital admissions. One concern is that with the suspension of routine dental care, more patients than usual could need admission for the management of acute dental infections that threaten the airway and require intensive care. This study is one of the first, along with a few others [8], to examine the impact of the wavelike COVID-19 pandemic on the emergency dental and oral and maxillofacial surgery services of a university hospital over time from January 2020 to 31 December 2020 in comparison with 2019.

## Materials and methods

In this retrospective study, all patients presenting to the dental and oral and maxillofacial emergency services of the Johannes Gutenberg-University Hospital Mainz, Germany in the COVID-19 pandemic year 2020 (1 January 2020 to 31 December 2020) were analysed (*n* = 3679). The patient collective from 2019 served as the comparison group (*n* = 4707). The study was approved by the local ethic committee of Rhineland-Palate (registration number 2020–15,530) and was conducted in accordance with the protocol and in compliance with the moral, ethical and scientific principles governing clinical research as set out in the Declaration of Helsinki of 1975 as revised in 1983. Outside regular opening hours, the dental emergency service was available from 17:00 to 22:00 on Mondays to Fridays and from 08:00 to 22:00 on weekends and public holidays. Outside regular opening hours, the oral and maxillofacial emergency service was available Mondays to Thursdays from 17:00 to the following day at 07:30, and continuously from Fridays at 13:00 to Mondays at 07:30.

According to the emergency service times, a list of all patients presenting during this period was created using the search filter function of the hospital system (SAP SE, Germany). Subsequently, each medical record of the total of 8386 included patients was screened for the following parameters and the information was recorded in a previously created Excel table. In each case, 1677 patient cases were processed by one of the five authors involved. After complete recording, all data were coded for statistical processing according to the groups described in M&M. Patient demographic characteristics such as gender, age, pregnancy, co-morbidities and specific long-term medications were recorded, as well as information on the reason (Table 1) and type (i.e. autonomous visit, referral, consultative, via rescue service) of presentation, diagnosis, diagnostic imaging (orthopantomography (OPT), cone beam computed tomography (CBCT), dental film, CT, sonography, MRI) and treatment. Co-morbidities of interest recorded were heart disease (e.g. previous myocardial infarction, arrhythmia, myocarditis, heart failure, coronary artery disease), hypertension, diabetes (type I and type II), any form of autoimmune disease, epilepsy, lung disease (asthma, chronic obstructive pulmonary disease (COPD), pulmonary fibrosis), congenital or acquired coagulopathies, as well as thyroid, kidney or liver disease. From March 2020, corona risk assessment was carried out on every scheduled or emergency patient. From March 2020 onwards, the COVID risk classification of each scheduled or emergency patient was carried out by means of a special questionnaire on personal travel history to risk regions/countries, clinical symptoms or previous contacts with COVID-positive tested persons or suspected cases in the personal environment. With regard to long-term medication, we focused on therapy-influencing drug groups such as oral anticoagulants, antiplatelet agents, dual and triple regimes of anticoagulation, antidiabetics and antihypertensives. The subdivision into diagnosis types/groups was as follows: conservative dental issues (e.g. acute pulpitis, periodontitis, tooth structure defects, loosened fillings, caries), trauma (e.g. anterior dental trauma, intra- and extraoral trauma, fractures of the midface, mandible or alveolar process), infections (e.g. submucosal abscess, periodontal abscess, lodge abscess, pericoronitis, maxillary sinus empyema, cervicofacial inflammations (e.g. phlegmon, superficial phlegmon, superinfected atheroma, infected wounds)), other disease (diseases of the oral mucosa (e.g. gingivitis, leukoplakia, erythroplakia, unclear tissue proliferation, mouth burning), defective orthodontic appliance, bone cyst, oroantral communication, sialadenitis, temporomandibular joint diseases (TMJ) in general, TMJ luxation, postoperative complications (e.g. wound dehiscence, wound infections, dry socket, swelling, pain), animal bites, sinusitis, cervicofacial emphysema, lymphadenopathy, prosthetic problems (e.g. denture pressure point, denture fracture, implant-associated problems (e.g. loosened cover screw or gingiva former)), cancer, scheduled follow-up presentations, bleeding (e.g. postoperative bleeding) and pain (craniofacial pain/discomfort with no clear aetiology, trigeminal neuralgia).

**Table 1:** Characteristics of patient-reported reasons for presenting during emergency hours

Patients’ reason for presentation
Pain
Accident
Orofacial swelling
Scheduled presentation for follow-up care
Dental conservative issues
• Loosened fillings (resin composite)
• Lost coronal seal (Cavit)
• Tooth structure defects
Tooth fractures
(Post) bleeding
Assault
Others
Orthodontic issues
• Loosened orthodontic devices
• Sharp-edged wire ends
Pus/fistula
Teething
Implant issues
• Loosened gingiva former or cover screw
• Loosened implant crown
Oral mucosal changes
Prosthetic issues
• Problems with prosthetic dentures and restorations
TMJ dislocation
Postoperative complications

Further, the diagnoses were grouped into emergent^+++^, urgent^++^ and non-urgent^+^ according to their need for treatment, following the classification scheme of the Scottish Dental Society. The different therapy measures were assigned to the following therapy groups for a better overview: splinting (i.e. using bony and dental-supported mandibulo-maxillary fixation, segmental wire-composite splints, titanium trauma splints, Schuchardt splints), dental conservation measures (i.e. resin composite filling, smoothing of sharp tooth or restoration edges), endodontic measures (i.e. trepanation, vital pulp extirpation, root canal treatments), surgical measures (i.e. extra-oral and/or intra-oral wound closure, haemostasis by means of vascular transection, mucoplasty, extraction of teeth or tooth remnants not worth preserving, extraoral incision and drainage of lodge abscesses), intraoral abscess incision and drainage, local haemostyptic haemostasis via tranexamic acid swab or silicone squeeze bite, conservative (symptomatic non-invasive) measures (i.e. prescription of oral painkillers, local cold application or soft-liquid food), follow-up measures (bandage or drainage change, suture removal, irrigation of an abscess cavity or maxillary sinus), TMJ repositioning, prescription of antibiotics, hospital admission and other isolated measures. More than one therapeutic intervention was recorded per patient case, where applicable. The epidemiological data on the development of COVID-19 infection figures in Germany were taken from the COVID-19 Dashboard (https://experience.arcgis.com/experience/478220a4c454480e823b17327b2bf1d4), the platform officially used by the Robert Koch Institute to provide the population with daily updates. Key figures on the worldwide COVID-19 situation are taken from statistical data on COVID-19 from the search engine platform Google (Google LLC, California, USA), whose sources include the free internet encyclopaedia Wikipedia, ministries of health or newspaper publishers such as the *New York Times*.

## Statistics

Raw data sets were saved in Excel sheets (Microsoft Corporation, Redmond, USA) and subsequently transferred into SPSS Statistics (version 26.0.0.0, MacOS X; SPSS Inc., IBM Corporation, Armonk, NY, USA). Data were expressed as mean (M), SD ± , minimum (min), maximum (max) and SEM. Normal distribution was checked using non-parametric Kolmogorov–Smirnov test (KS test) and results were analysed for statistical significance by the use of ANOVA = ^(#)^, unpaired non-parametric Mann–Whitney *U* tests = ^($)^ and Students’ *t* test = ^(^*^)^. *P* values of ≤ 0.05 were termed significant. Line and bar charts were used for illustration purposes. Timeline graphics were created with Office Timeline Pro (Microsoft Corporation, Redmond, USA).

## Results

### Dental emergency service—characteristics before and during COVID-19 pandemic

**Fig. 1:**
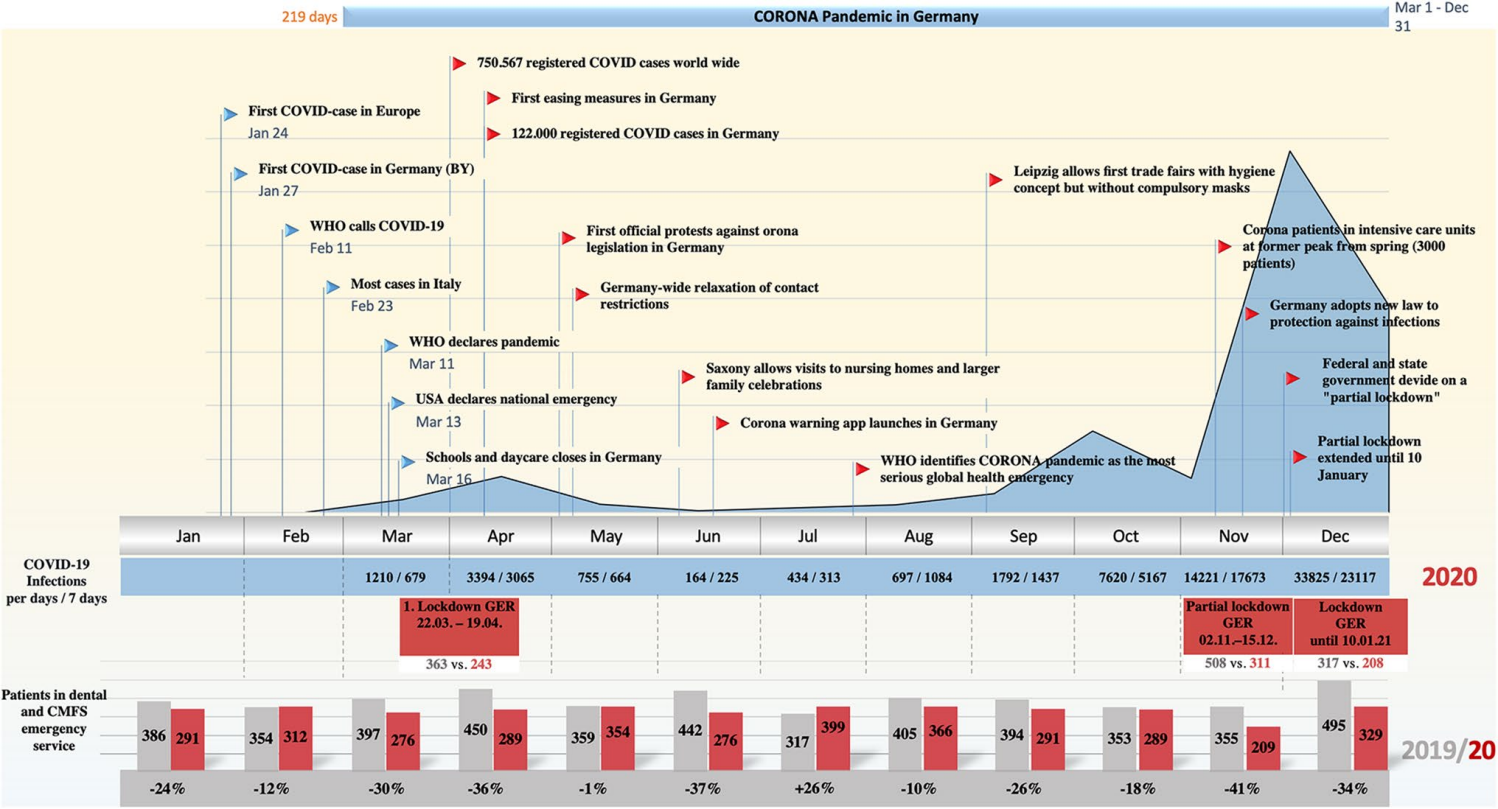
Timeline shows the COVID-19 year 2020 and the monthly development of the nationwide infection figures, in correlation with the monthly patient numbers of the dental and CMFS emergency services (red), taking into account the nationwide public health restrictions. The key figures from 2019 are shown in grey for comparison

**Fig. 2:**
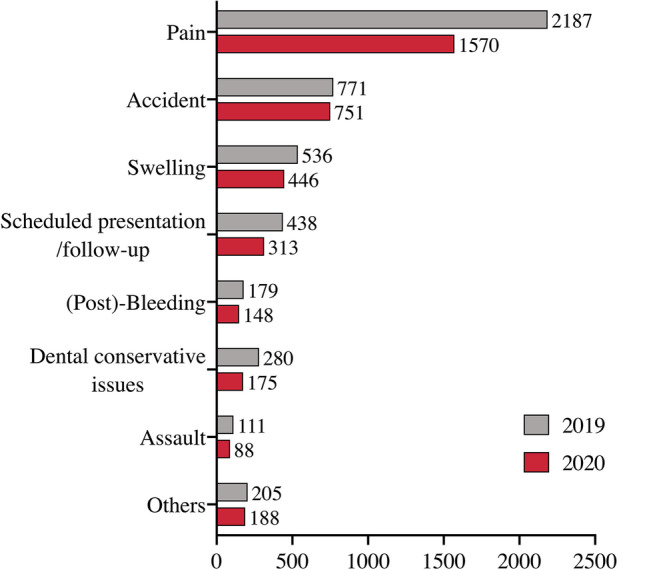
Bar chart shows the distribution of patient-side reasons for presentation in dental and maxillofacial emergency services in comparison of the years 2019 (grey) and 2020 (red)

In 2020, a total of 3679 patients (female = 1675 and male = 2004) presented from 1 January to 31 December. In 2019, a total of 4707 patients (female = 2121 and male = 2586) presented through the University Hospital’s emergency dental service during the same period. The average patient age in 2020 was 37.6 (min 0, max 96) and 37.2 years (min 0, max 98) in 2019. The number of emergency presentations in 2020 decreased by a total of 1026 patients (22%) compared with 2019. Broken down by calendar month, a similar picture emerges in percentage terms when comparing the calendar years 2019 and 2020 (Table 2). However, a direct comparison between 2019 and 2020 showed a significant reduction in patient presentations in 2020 (Table 2). In relation to the public restriction measures within the framework of the nationwide lockdown intervals, there was a clear decrease in presentations to the dental and oral and maxillofacial surgery emergency services at Mainz University Hospital up to 39% during the partial lockdown period from 2 November to 15 December 2020 (Table 3). Figure 1 provides an overview of the COVID-19 pandemic year 2020, the development of COVID-19 infection cases as well as the globally adopted measures and the restrictions on public life in Germany. The gender distribution did not differ relative to the number of patients when comparing 2019 and 2020. The same applied to the patients’ age distribution and the frequency of visits depending on the weekday. The daytime of the emergency presentation was also similar, with a prime-time period between noon and midnight (Table 4). The most common (> 95%) reasons for visits in the dental and CMFS emergency service were pain, accidents with facial trauma, swelling, scheduled presentations/follow-up, dental conservative issues, post-bleedings, assaults and others (Fig. 2).

**Table 2:** Trend in patient numbers in dental and craniomaxillofacial emergency services by month in comparison between 2019 und 2020

Month	Year 2019 (%)	Year 2020 (%)	Difference 2019 vs. 2020
January	386 (8.2)	290 (7.9)	− 25%
February	354 (7.5)	312 (8.5)	− 12%
March	397 (8.4)	272 (7.4)	− 31%
April	450 (9.6)	293 (8)	− 35%
May	359 (7.6)	354 (9.6)	− 1%
June	442 (9.4)	277 (7.5)	− 37%
July	317 (6.7)	399 (10.8)	+ 26%
August	405 (8.6)	366 (9.9)	− 10%
September	394 (8.4)	291 (7.9)	− 26%
October	353 (7.5)	289 (7.9)	− 18%
November	355 (7.5)	209 (5.7)	− 41%
December	495 (10.5)	329 (8.9)	− 33%
**Total**	**4707**	**3679**	** − 22%**

**Table 3:** Trends in dental and craniomaxillofacial emergency services patient numbers in 2019 and 2020, grouping into defined time periods according to federal restriction measures in 2020 and applied these on 2019 for better comparability

Time period	Year 2019 (%)	Year 2020 (%)	Difference 2019 vs. 2020 (%)
01.01–21.03 (Pre-LD)	1024 (21.8)	827 (22.5)	− 19
22.03–19.04 (LD #1)	363 (7.7)	243 (6.6)	− 33
20.04–01.11 (IM)	2495 (53.0)	2092 (56.8)	− 16
02.11–15.12 (Part.LD)	508 (10.8)	311 (8.4)	− 39
16.12–31.12 (LD #2)	317 (6.7)	206 (5.6)	− 34

**Table 4:** Characteristics of dental and oral and maxillofacial surgery emergency service patients compared in 2019 and 2020

Variables	Year 2019 (%)	Year 2020 (%)					
	Count	Count	Pre-LD	LD #1	IM	Part-LD	LD #2
Total	4707	3679	827 (22.5)	243 (6.6)	2092 (56.8)	311 (8.4)	206 (5.6)
Gender							
Female	2121 (45.1)	1675 (45.5)	350 (42.3)	114 (46.9)	971 (46.4)	149 (47.9)	91 (44.2)
Male	2586 (54.9)	2004 (54.5)	477 (57.5)	129 (53.1)	1121 (53.6)	162 (52.1)	115 (55.8)
Age group (years)							
0–3	228 (4.8)	189 (5.1)	34 (4.1)	18 (7.4)	114 (5.4)	13 (4.2)	10 (4.9)
4–14	481 (10.2)	392 (10.7)	81 (9.8)	28 (11.5)	232 (11.1)	34 (10.9)	17 (8.3)
15–30	1299 (27.6)	948 (25.8)	215 (26)	56 (23)	555 (26.5)	71 (22.8)	51 (24.8)
31–65	2171 (46.1)	1698 (46.2)	399 (48.2)	114 (46.9)	920 (44.0)	165 (53.1)	100 (48.5)
66–80	383 (8.1)	311 (8.5)	70 (8.5)	19 (7.8)	185 (8.8)	16 (5.1)	21 (10.2)
> 80	145 (3.1)	141 (3.8)	28 (3.4)	8 (3.3)	86 (4.1)	12 (3.9)	7 (3.4)
Weekday							
Monday	376 (8)	293 (8)	67 (8.1)	33 (13.6)	160 (7.6)	19 (6.1)	14 (6.7)
Tuesday	361 (7.7)	223 (6.1)	47 (5.7)	11 (4.5)	136 (6.5)	21 (6.8)	8 (3.8)
Wednesday	368 (7.8)	269 (7.3)	78 (9.4)	10 (4.1)	152 (7.3)	20 (6.4)	9 (4.3)
Thursday	354 (7.5)	324 (8.8)	64 (7.7)	22 (9.1)	164 (7.8)	22 (7.1)	52 (25)
Friday	516 (11)	482 (13.1)	77 (9.3)	37 (15.2)	291 (13.9)	46 (14.8)	31 (14.9)
Saturday	1411 (30)	1134 (30.8)	295 (35.7)	63 (25.9)	623 (29.8)	102 (32.8)	51 (24.5)
Sunday	1321 (28.1)	956 (26)	199 (24.1)	67 (27.6)	566 (27.1)	81 (26)	43 (20.7)
Daytime							
Midnight–6:00	271 (5.8)	222 (6.0)	51 (6.2)	8 (3.3)	148 (7.1)	9 (2.9)	6 (2.9)
6:01–noon	1065 (22.6)	797 (21.7)	185 (22.4)	58 (23.9)	428 (20.5)	66 (21.2)	60 (29.1)
12:01–18:00	1623 (34.5)	1329 (36.1)	299 (36.2)	97 (39.9)	730 (34.9)	120 (38.6)	83 (40.3)
6:01–23:59	1748 (37.1)	1331 (36.2)	292 (35.3)	80 (32.9)	786 (37.6)	116 (37.3)	57 (27.7)

### Disease types, diagnoses and urgency

**Fig. 3:**
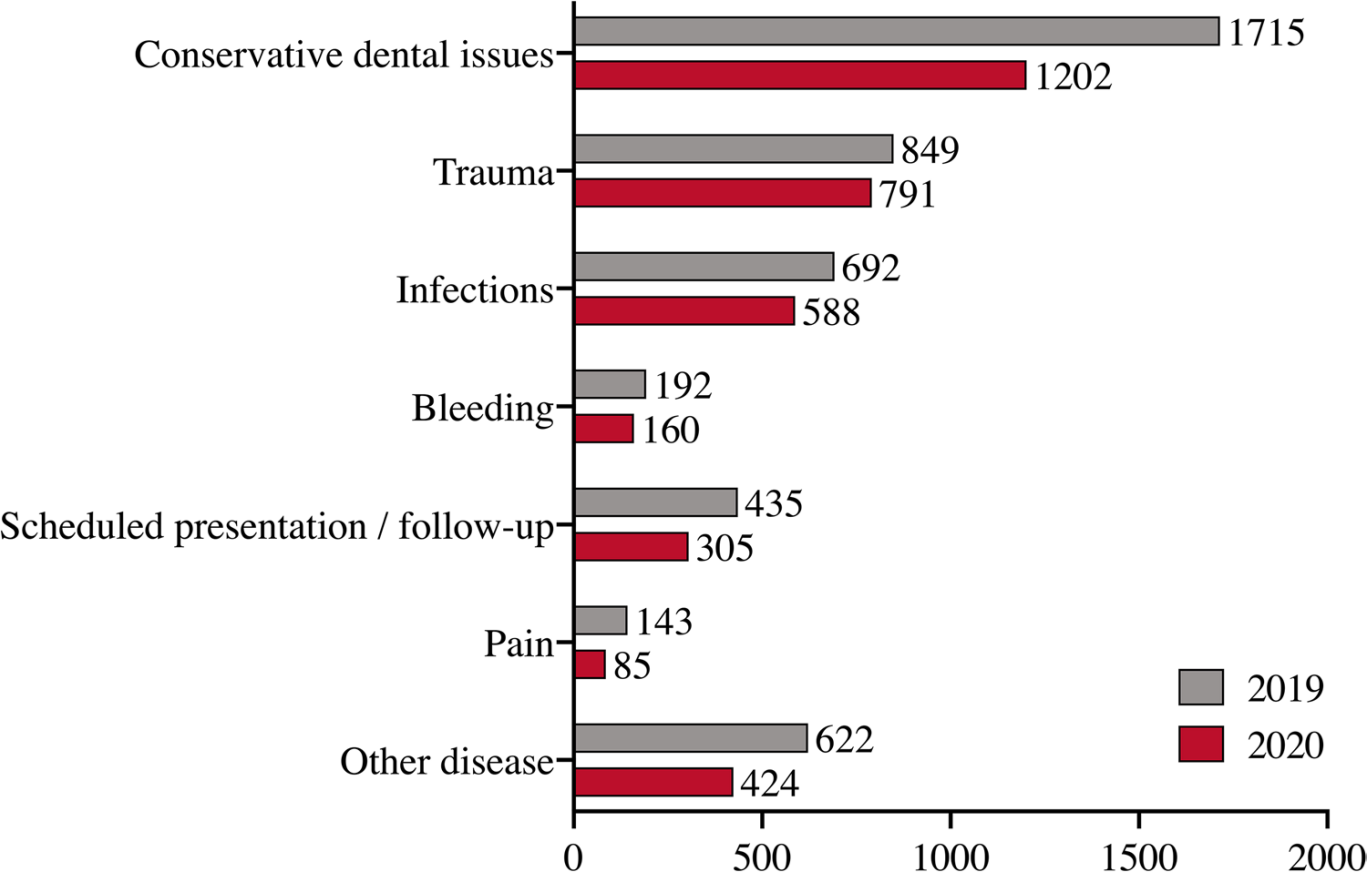
Bar chart showing the distribution of diagnosis types in dental and maxillofacial emergency services comparing 2019 (grey) and 2020 (red)

The most common diagnosis for presentation in both 2019 (36.4%) and 2020 (32.7%) were general dental conservative pathologies including caries, crown- or root fractures, acute pulpitis, or apical periodontitis. When comparing 2019 and 2020, there was a significant decrease in frequencies (*p* = 0.003). Orofacial hard and soft tissue traumas including dental trauma, lacerations, central and centrolateral midface and mandibular fractures were second most common in 2019 (18%) and 2020 (21.5%), whereby its total number decreased significantly in 2020 (*p* < 0.001). Infectious-inflammatory pathologies were the third most common type of diagnosis in 2019 (14.7%) and 2020 (16%), with little change in incidence (*p* = 0.106), followed by the diagnosis type “others” (13.2% and 11.5%), scheduled follow-up presentations (9.2% and 8.3%), bleedings (3.3% and 3.5%), and facial pain (3% and 2.3%; Table 5, Fig. 3). Although less in total, trauma (+ 3.5%) and infectious pathologies (+ 1.3%) have increased proportionately in 2020 when compared to 2019. Among cases with post-bleeding following oral surgery, the proportion taking non-vitamin K antagonist oral anticoagulants (NOACs) was 20.4% in 2019 (*n* = 32/157) and 25% (*n* = 32/129) in 2020. Continuous medication with vitamin K antagonist anticoagulants (OACS) was present in 14% (22/157) in 2019 and in 18.6% (24/129) in 2020. Detailed information on the frequency distribution of the diagnoses is given in Table 5. Further, it was shown that the proportion of emergent presentations^+++^ increased by 6.3% in COVID-19 year 2020, and the proportion of urgent^++^ and non-urgent^+^ presentations decreased correspondingly by 4.2% and 2.1%, respectively (Table 6).

**Table 5:**
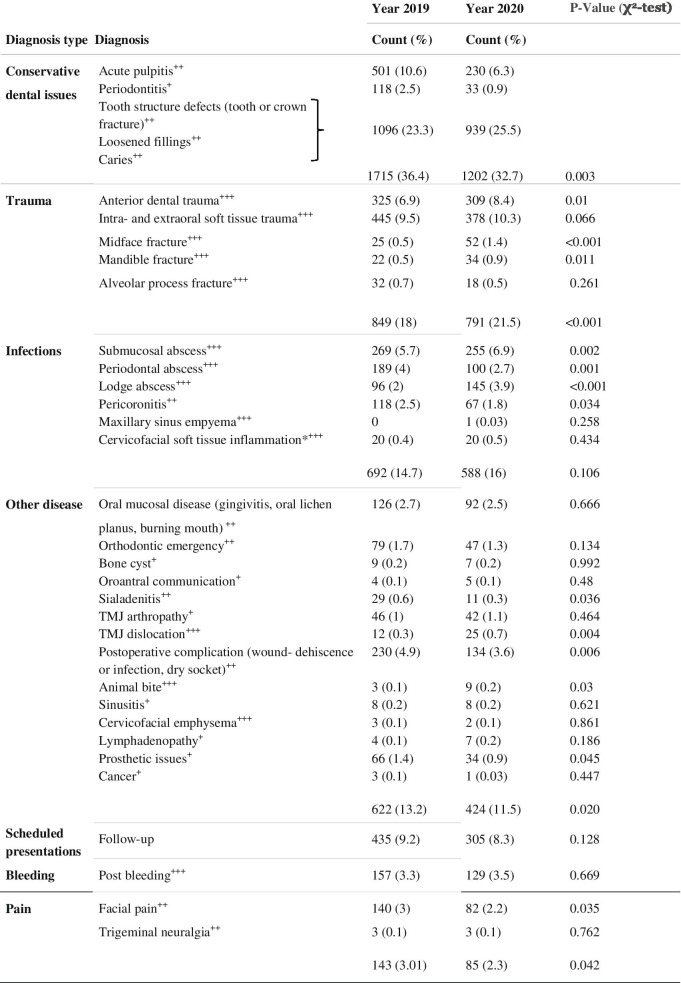
Diagnoses made by the physician/dentist for emergency presentation

Cervicofacial infections (*) include cutaneous abscesses, boils, infected atheromas, phlegmon and erysipelas. Assignment to one of the urgency groups is indicated by the superscript + signs (+ +  +  = emergent; +  +  = urgent; +  = non-urgent)

**Table 6:** Number and proportion (%) of cases grouped by urgency and presented comparing 2019 and 2020

Urgency	Year 2019 (%)	Year 2020 (%)	*P* value (*χ*^2^ test)
Emergent^**+++**^	1633 (34.7)	1508 (41)	< 0.001
Inpatient admission	45 (2.8)	129 (8.6)	
Urgent^**++**^	2174 (46.2)	1545 (42)	< 0.001
Inpatient admission	5 (0.2)	8 (0.5)	
Non-urgent^**+**^	900 (19.1)	625 (17)	0.012
Inpatient admission	–	7 (1.1)	
**Total**	4707 50 (1.1)	3679 144 (3.9)	< 0.001

Pre-LD = time period prior to the first lockdown (LD #1) in Germany; IM = intermediate period between first and partial lockdown (Part.LD); LD #2 = second lockdown in Germany.

### Lockdown periods

**Fig. 4:**
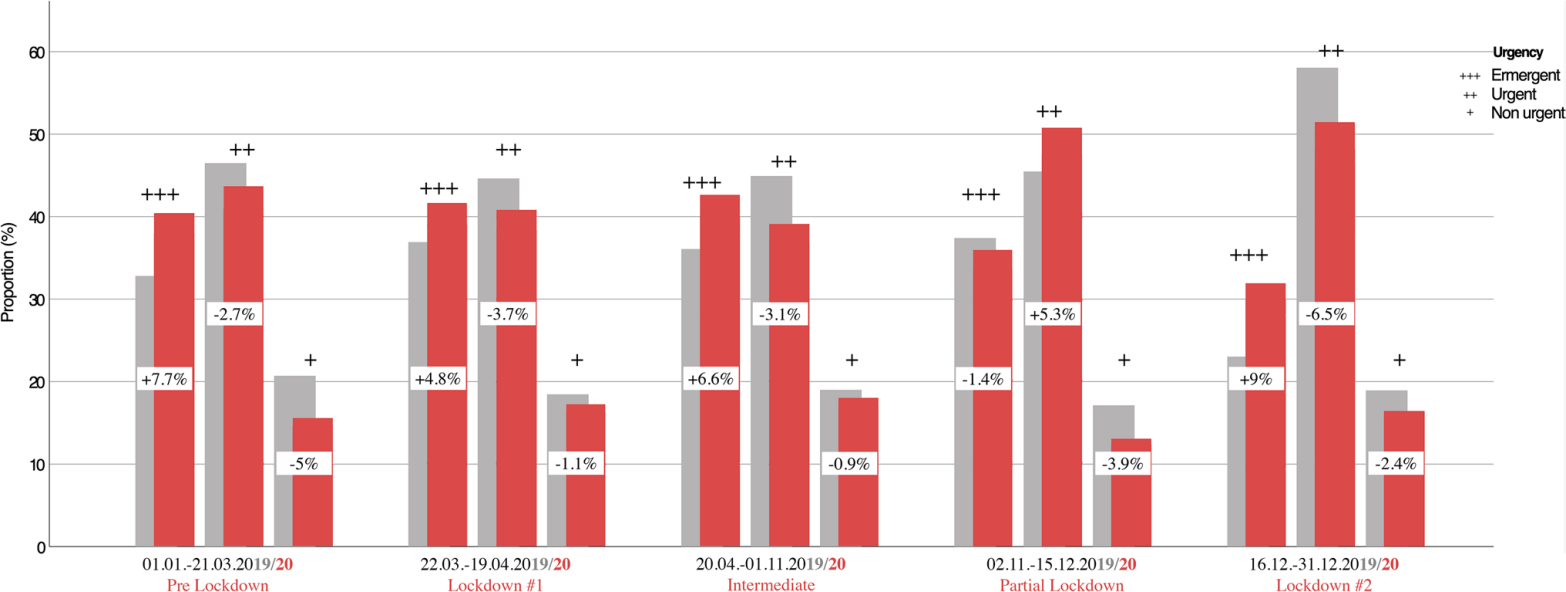
Bar chart shows the distribution of cases classified as emergency (+ + +), urgent (+ + +) and non-urgent ( +) in correlation to the federal lockdown periods comparing 2019 (grey) and 2020 (red)

In correlation with the public restrictions in 2020, except for 2 November through 15 December, there was an increase in cases actually defined as emergent^+++^ with a concomitant decrease in urgent^++^ and non-urgent^+^ presentations during emergency hours (Fig. 4). This, in turn, was also reflected in the number of inpatient admissions during emergency service hours, when the number of 50 (1.1%) inpatient admissions during emergency service in 2019 more than doubled to 144 (3.9%) cases in 2020, more than tripling the proportion (Table 6). This difference was statistically significant at *p* < 0.001. Here, the group of cases classified as emergent^+++^ among inpatients admitted to the hospital accounted for 90% (45/50) in 2019 and 89.6% (129/144) in 2020, respectively. Cases classified as urgent^++^ based on diagnosis each accounted for 10% (5/50) in 2019, as well as urgent^++^ and non-urgent^+^ each accounted for 5.6% (8/144) and 4.9% (7/144) in 2020.

### Therapeutic measures

Table 7 shows the treatments performed during the emergency service hours and their frequencies in 2019 and 2020. Compared with 2019, significantly more surgical procedures, scheduled follow-up visits, and TMJ repositioning were performed in 2020, as well as consults co-assessed and oral antibiotics prescribed (Table 7). For all other measures recorded (dental and or jaw splinting), dental conservative as well as endodontic measures, abscess incisions, local haemostasis, conservative (symptomatic non-invasive) measures (e.g. prescription of pain medication, local cold or soft-liquid food) or others, there was no significant difference between 2019 and 2020.

**Table 7:** Therapeutic measures and their frequency compared between 2019 and 2020

	Year 2019	Year 2020	*P* value (*χ*^2^ test)
Therapy	Count (%)	Count (%)	
Tooth/jaw splinting	84 (1.8)	84 (2.3)	0.11
Dental conservation measures	615 (13.1)	454 (12.3)	0.32
Endodontic measures	628 (13.3)	452 (12.3)	0.15
Surgical measures	488 (10.4)	452 (12.3)	0.006
Abscess incision	471 (10)	379 (10.3)	0.66
Local haemostatic measures	140 (3)	82 (2.2)	0.97
Conservative (symptomatic non-invasive) measures	1744 (37.1)	1367 (37.2)	0.92
Consultative co-assessment	42 (0.9)	62 (1.7)	0.001
Follow-up care	451 (9.6)	305 (8.3)	0.02
Other measures*	138 (2.9)	122 (3.3)	0.31
TMJ reposition	12 (0.3)	24 (0.7)	0.006
Prescription of antibiotics	447 (9.5)	562 (15.3)	0.03

Other measures ^(*)^ include performing temporary refixation of loosened crowns and partial crowns, grinding in dentures that cause pressure points, or removing sharp wire ends of orthodontic appliances.

## Discussion

This is the first study to examine the impact of the COVID-19 pandemic on the emergency dental and maxillofacial services of a German university hospital in 2020 compared to the previous year 2019 in relation to the German federal restriction measures (lockdown periods). Limitations of the present study are the limited transferability of the data to other countries, which is not least due to the country-specific regulations on the health system under pandemic conditions, as well as the inherent limitations of retrospective studies. This includes the evaluation of partially incomplete data sets, as the definition of specific parameters of interest is missing, unlike a prospective study design, and results may be influenced by parameters that were not recorded at all [9, 10]. More years could have been used as a reference period, as the comparison year 2019 could be an exception. However, this was not done, taking into account an earlier study on the emergency dental service of our university hospital in the years 2010–2013 [2]. The epidemiological data included patient characteristics, reasons for presentation and times, as well as diagnoses made and treatments carried out. This revealed that the number of emergency service visits in 2020 decreased significantly by 22% compared to 2019. Broken down by month, there were proportional peaks of up to − 41% in November 2020, but also a 26% increase in visits in July 2020. The evaluation by defined time periods, which corresponded to the periods of the federal public life restriction measures, compared to the same periods in 2019, showed a decrease in emergency service visits of 33% during the first lockdown (22 March 2020 to 19 April 2020), by 39% during the partial lockdown (2 November 2020 to 15 December 2020) and 34% during the second lockdown from 16 December 2020 to 31 December 2020. This, as well as the phenomenon of a proportionally significant increase in individual types of diagnosis (e.g. trauma and infectious pathologies) with a simultaneous decrease in the total number of emergency presentations, was consistent with the study results of the Italian study group around Cagetti et al. [11]. In contrast, a Swiss study showed an increase in daily case numbers during the lockdown periods, but only the pre-COVID period 2020 served as a reference [8]. Regarding the number of odontogenic infections, an American study by Johnson et al. showed a numerical decrease over the limited period from March to June, but a proportional increase compared to the years 2017–2019 [12]. Overall, there is currently little epidemiological data on the use of dental emergency service in connection with the COVID-19 pandemic. Most of the studies published so far are from 2020, describe the situation outside Europe (East Asia or South America) [13, 14] and only highlight parts of the year [15], so they do not provide information on the situation in Europe, nor the characteristics of emergency service visits over the course of the COVID-19 infection waves and the associated constraints on public life [14, 16–18]. The decrease in the total number of patients due to the COVID-19 pandemic in 2020 shown in this study is consistent with the findings of other studies [11, 19]. However, the varying availability of the emergency units should be taken into account, which has an influence on the number of patients recorded [11, 17, 20]. The isolated increase of 26% in emergency service visits in July 2020 compared to the previous year observed in this study also seems to follow a certain systematic approach, taking into account recent study results from Italy. In this regard, the group of authors around Cagetti et al. showed that there was an increase in emergency service requests in the period after general relaxations of COVID-related restrictions on public life [11]. An explanation for this is the general uncertainty of the population and (dental) healthcare professionals, which is due not least to the fear of infections, but also to non-existent or controversial recommendations by professional societies and governments, and which has led to a delayed use of a medical examination with aggravation of the diagnostic severity and urgency. In detail, these were inconsistent regulations on the maintenance of outpatient care in the field of dentistry and general medicine, as well as the definition of urgently necessary and avoidable treatments. An example of this is the risk of aerosol formation in the context of dental treatments, which was discussed at the beginning but is difficult to avoid in theory and practice. Contrary to the fear of self-infection, a recent study from India on paediatric dental treatments during the COVID-19 pandemic could not prove any infection of dental staff in the course of aerosol-generating therapy measures [21]. At the beginning of the pandemic, orthodontic treatments should therefore be continued in Germany due to almost no aerosol formation but indicated continuation of treatments already started, whereas periodontal therapies or prophylactic treatments should be suspended. This theory is supported by the results of studies that have shown a direct link between a decrease in dental treatments and patient fear of SARS-CoV-2 infection [22–24]. One possible solution is seen in open doctor–patient communication, explaining to the patient the measures taken by the treating institution to minimise the incidence of infection, and highlighting the positive and negative consequences of treatment [25]. Despite fewer overall visits during emergency hours in 2020, our study revealed that there was an increase in the proportion of cases defined as emergent^+++^ (+ 6%), with a simultaneous decrease in urgent^++^ (− 4.2%) and non-urgent^+^ presentations (− 2.1%). This was in line with the increasing proportion of patients who were further admitted for stationary care following emergency presentation as it tripled in 2020 compared with 2019, with almost 90% of cases classified as “emergent” (*p* < 0.001).

## Conclusions

The year 2020 has not only shown the imperative need to secure a dental as well as maxillofacial surgery emergency service system, but equally the importance of stable basic dental and maxillofacial surgical care in times of pandemic. However, this requires the provision of uniform, cross-national recommendations for action and government regulations by relevant professional societies and politics.

## Data Availability

The datasets used and/or analysed during the current study are available from the corresponding author on request.

## Declarations

### Authors’ information

The results of the present study are part of the doctoral thesis of the co-author and student of the University of Mainz, M.P., and as such will be made publicly available as part of the doctorate.
